# Tacrolimus-Based versus Cyclosporine-Based Immunosuppression in Hepatitis C Virus-Infected Patients after Liver Transplantation: A Meta-Analysis and Systematic Review

**DOI:** 10.1371/journal.pone.0107057

**Published:** 2014-09-08

**Authors:** Zhenmin Liu, Yi Chen, Renchuan Tao, Jing Xv, Jianyuan Meng, Xiangzhi Yong

**Affiliations:** 1 Department of Periodontology and Oral Medicine, College of Stomatology, Guangxi Medical University, Nanning, Guangxi, China; 2 Department of Hepato-biliary Surgery, First Affiliated Hospital of Guangxi Medical University, Nanning, Guangxi, China; Centro de Investigación en Medicina Aplicada (CIMA), Spain

## Abstract

**Background:**

Most liver transplant recipients receive calcineurin inhibitors (CNIs), especially tacrolimus and cyclosporine, as immunosuppressant agents to prevent rejection. A controversy exists as to whether the outcomes of hepatitis C virus (HCV)-infected liver transplant patients differ based on the CNIs used. This meta-analysis compares the clinical outcomes of tacrolimus-based and cyclosporine-based immunosuppression, especially cases of HCV recurrence in liver transplant patients with end-stage liver disease caused by HCV infection.

**Methods:**

Related articles were identified from the Cochrane Hepato-Biliary Group Controlled Trials Register, the Cochrane Central Register of Controlled Trials (CENTRAL) in the Cochrane Library, Medline, and Embase. Meta-analyses were performed for the results of homogeneous studies.

**Results:**

Nine randomized or quasi-randomized controlled trials were included. The total effect size of mortality (RR = 0.98, 95% CI: 0.77–1.25, *P* = 0.87) and graft loss (RR = 1.05, 95% CI: 0.83–1.33, *P* = 0.67) showed no significant difference between the two groups irrespective of duration of immunosuppressant therapy after liver transplantation. In addition, the HCV recurrence-induced mortality (RR = 1.11, 95% CI: 0.66–1.89, *P* = 0.69), graft loss (RR = 1.62, 95% CI: 0.64–4.07, *P*  = 0.31) and retransplantation (RR = 1.40, 95% CI: 0.48–4.09, *P* = 0.54), as well as available biopsies, confirmed that histological HCV recurrences (RR =  0.92, 95% CI: 0.71–1.19, *P* = 0.51) were similar.

**Conclusion:**

These results suggested no difference in posttransplant HCV recurrence-induced mortality, graft loss and retransplantation, as well as histological HCV recurrence in patients treated with tacrolimus-based and cyclosporine-based immunosuppresion.

## Introduction

Hepatitis C virus (HCV) infection constitutes a serious challenge to global health, and accounts for the loss of approximately 12,111,000 Disability-Adjusted Life Years (DALYs) [1]. Also, end-stage liver diseases caused by HCV infection are the leading indication for orthotropic liver transplantation. Unfortunately, recurrent hepatitis C is universal, resulting in accelerated progression to cirrhosis with 5 years of transplantation for 10%–30% of patients [2] and causing graft loss as well as the need for retransplantation. Thus, good control of the virus before and after transplantation to alleviate recurrence, as well as accessing sufficient numbers of liver grafts, are both enormous challenges in liver transplantation. Attempts to prevent reinfection and rejection via antiviral treatment with a combination of immunosuppressant regimens gave promising results [3]. However, options for antiviral therapy are limited and are associated with a significant side-effect profile mainly caused by pegylated interferon and ribavirin therapy [4]; future interferon-free antiviral therapies will be more efficacious with fewer side effects. In addition, the dosage, duration, and composition of immunosuppressant regimens vary in different liver transplant centers.

Cyclosporine has been used as an effective immunosuppressant after liver transplantation since 1983, showing significant clinical advances in graft survival and patient survival in organ transplants [5], [6]. Subsequently, tacrolimus was found to have a mechanism of action in inhibition of calcineurin phosphatase (CNI) consistent with that of cyclosporine, even though they bind different intracellular immunophilins [7]. As for better efficacy with respect to reduced mortality and graft loss with immunosuppressant therapy, tacrolimus has been the primary immunosuppressant agent. However, cyclosporine has been reported to have antiviral effects by suppressing the replication of the hepatitis C virus [8], [9] and increasing the chance of a sustained virological response after transplantation [10], an effect that was not detected with tacrolimus [11]. Controversy exists as to whether the clinical outcomes of HCV infection-related liver transplants differ depending on the types of CNIs used. However, immunosuppression is a major factor responsible for accelerated recurrence and faster progression of recurrent HCV infection [12]. A meta-analysis [13] reported patient and graft survivals in HCV-positive liver transplant patients were similar regardless of the CNIs selected as the basic immunosuppressant. However, few studies focused on the differences in efficacy between tacrolimus and cyclosporine which might affect HCV recurrence. Therefore, the purpose of our meta-analysis was to evaluate clinical outcomes, especially cases of HCV recurrence in liver transplantation comparing tacrolimus-based and cyclosporine-based immunosuppression.

## Patients and Methods

### Inclusion criteria

The inclusion criteria were: 1) Randomised and quasi-randomised controlled trials which compared tacrolimus with cyclosporine solution (Sandimmune) or cyclosporine microemulsion (Neoral) as immunosuppressive therapy in patients with end-stage liver disease caused by HCV infection who underwent a primary liver transplant; 2) Trials in which a group of HCV patients were considered as a subgroup, and results reported the variables of interest in our studies, with sufficient data for calculating the risk ratio (RR) with 95% confidence interval (CI). 3) A minimum of one year duration of follow-up. If the results of interest from patients in a clinical trial were reported more than once, data from the publication with the longest follow-up were extracted. In addition, studies on patients who underwent multi-organ transplantation or had previously received a liver transplant were excluded.

### Search strategy

We performed an electronic search of the following databases: The Cochrane Hepato-Biliary Group Controlled Trials Register (up to March 2014), the Cochrane Central Register of Controlled Trials (CENTRAL) in the Cochrane Library (up to March 2014), Medline (1948 to March 2014), and Embase (1980 to March 2014). Key words used in the search were: “tacrolimus” or “FK506”, and “cyclosporine” or “Neoral” or “calcineurin inhibitors” or “cyclosporine A (CyA)”, as well as “liver transplantation”. In addition, hepatitis C or HCV-related and HCV recurrence were included.

### Data extraction

Standardized forms were designed for data extraction; two investigators entered the data on patient demographics, duration of follow-up, methodology, blood concentration of tacrolimus and cyclosporine in the first three months and in the 12th month of immunosuppressant therapy, combination regimens, and occurrence of the following outcomes: mortality, graft loss, histological HCV recurrence; and mortality, graft loss as well as retransplantation due to HCV respectively. Any inconsistencies were addressed by further discussion.

### Statistical analysis

Assessment of effect sizes and heterogeneity were performed using Review Manager 5.2 software. Pooled RR and 95% CIs were calculated for categorical outcomes using fixed effects models; if no significant heterogeneity was present, random effects models were used. Heterogeneity among trials was assessed by Cochran's Q-statistic and I^2^ index, when the P-value for heterogeneity was <0. 1 or I^2^>50%, significant heterogeneity was detected; if statistic heterogeneity was present in meta-analysis, sensitivity analysis was conducted by subsequent exclusion of the single study with the highest weight to assess the validity of outcome.

Publication bias including funnel plot and Egger's test was performed using STATA version 10.0.

## Results

### Study description

**Figure 1:**
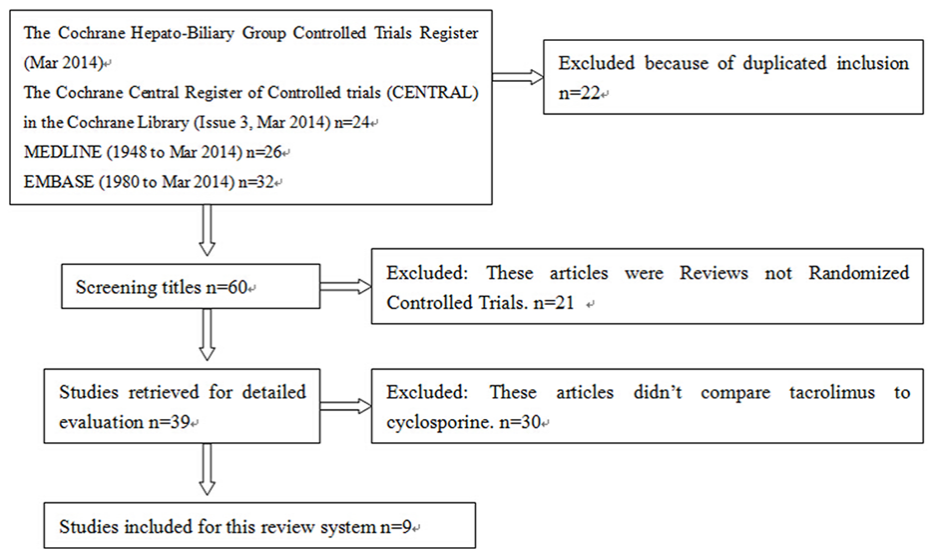
Flow chart of the selection process for including articles.

The search was performed in March 2014, and 82 studies were found in four public databases. Nine randomized and quasi-randomized controlled trials allocated 1180 participants of whom 570 were randomized to tacrolimus-based and 610 to cyclosporine-based. Studies were excluded if they were not originally designed to compare tacrolimus to cyclosporine. Additionally, duplicated publication or non-randomized controlled trials were also excluded (Fig 1). Across these nine studies, all participants received a primary liver transplantation with end-stage liver disease caused by HCV infection. HCV recurrence was measured and assessed using liver biopsy. The characteristics of randomized and quasi-randomized trials included in the systematic review are shown in Table 1.

**Table 1:** Characteristics of randomized and quasi-randomized trials included in the systematic review.

			Tacrolimus concentration(ng/ml)		Cyclosporine concentration(ng/ml)				
Trials	Methods	N	0–3 month	1 year	0–3 month	1 year	Anti-proliferative agent	Steroids	Duration of follow-up
Levy 2014 [25]	Multicenter, randomized, open-label study	Tac n = 182 CsA n = 169	NS	NS	NS	NS	MMF/AZA	Not stated detail regimen of steroids OR steroids-free	12 months
Berenguer 2010 [24]	Pseudo-randomized controlled trial	Tac n = 117 CsA n = 136	5–15	3–10	150–350	100–150	MMF	Methyl-prednisolone &prednisone	7 years
Shenoy 2008 [18]	Single-center, prospective, randomized trial	Tac n = 14 CsA n = 18	8–12	5–10	800–1200	600–1000	MMF	Methyl-prednisolone &prednisone	12 months
Villamil 2006 [26]	Multicenter open-label randomized study	Tac n = 48 CsA n = 47	NS	NS	NS	NS	AZA	Not stated the detail of Steriods	34–37 months
Levy 2006 [17]	Multicenter, randomized, open-label, parallel-group, prospective study	Tac n = 85 CsA n = 88	5–15	5–10	800–1200	500–700	AZA	Methyl-prednisolone &prednisone	12 months
Martin 2004 [37]	Prospective, randomized, multicenter, open-label study	Tac n = 38 CsA n = 41	10–12	5–10	200–250	100–250	AZA	Prednisone	12 months
Zervos 1998 [31]	Randomized, prospective study	Tac n = 25 CsA n = 24	15	NS	300–400	NS	NS	Methyl-prednisolone &prednisone/Prednisone	18 months
Wiesner 1998 [38]	Randomized comparative open-label study	Tac n = 57 CsA n = 56	10–25	10–12	250–400	200–300	AZA	Methyl-prednisolone	5 years
Mueller 1995 [39]	Single-center, prospective, randomized trial	Tac n = 17 CsA n = 18	NS	NS	NS	NS	NS	Methyl-prednisolone	12 months

NS = not stated.

MMF = mycophenolate mofetil; AZA = azathioprine.

### Meta-Analysis of treatment efficacy

#### Mortality

**Figure 2:**
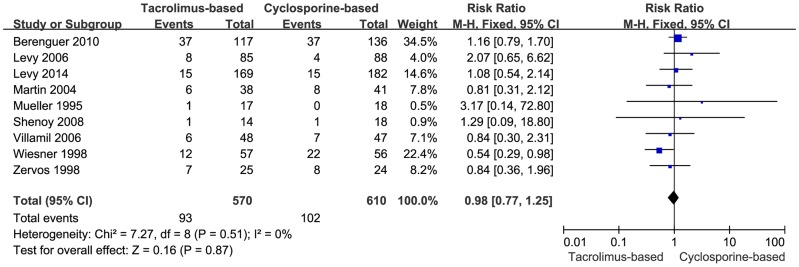
Forest plot of mortality comparing tacrolimus-based to cyclosporine-based immunosuppresant group.

By virtue of our selection criteria, mortality was reported in all of the included trials. There was no significant statistical difference in mortality between the two groups in the fixed-effects model (RR = 0.98, 95% CI: 0.77–1.25, *P* = 0.87) (Fig 2). Moreover, no heterogeneity among the included trials (*P* = 0.51, I^2^ = 0%) was observed.

#### Mortality due to HCV recurrence

**Figure 3:**
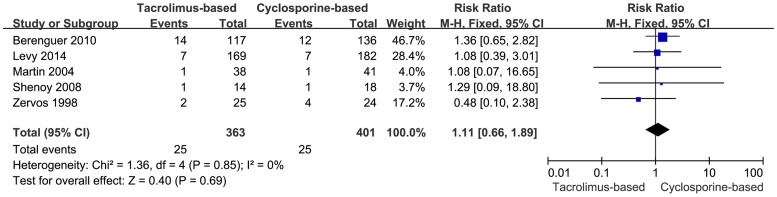
Forest plot of mortality due to HCV recurrence comparing tacrolimus-based to cyclosporine-based group.

Mortality due to HCV recurrence was reported in five trials. The total effect size of mortality due to HCV recurrence was similar (RR = 1.11, 95% CI: 0.66–1.89, *P* = 0.69) (Fig 3) in fixed-effects model. Moreover, no heterogeneity among the included trials (*P* = 0.85, I^2^ = 0.0%) was observed.

#### Graft loss

**Figure 4:**
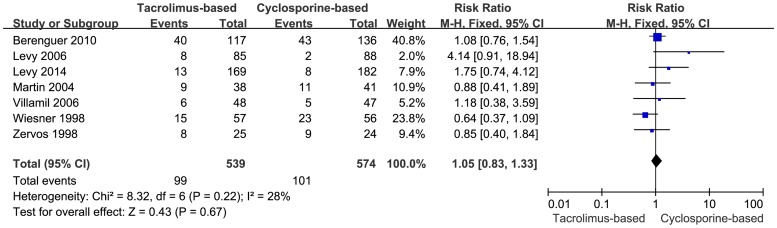
Forest plot of graft loss comparing tacrolimus-based to cyclosporine-based group.

Graft loss was reported in seven trials. A meta-analysis using fixed-effects model was performed with respect to graft loss, but the difference was too small to reach statistical significance (RR = 1.05, 95% CI: 0.83–1.33, *P* = 0.67) (Fig 4). Moreover, moderate inconsistencies existed among the trials (*P* = 0.22, I^2^ = 28%).

#### Graft loss due to HCV recurrence

**Figure 5:**
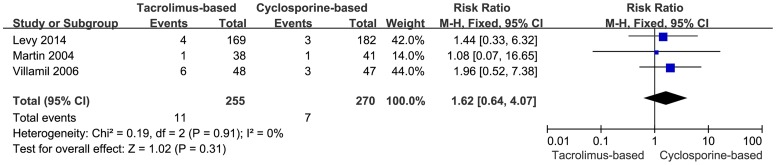
Forest plot of graft loss due to HCV recurrence comparing tacrolimus-based to cyclosporine-based group.

Data on graft loss due to recurrent HCV was only available in three trials. Eleven of the 255 patients in the tacrolimus-based group compared to 7 of 270 in the cyclosporine-based group lost their grafts owing to HCV recurrence. The difference did not reach statistical significance in the fixed-effects model (RR = 1.62, 95% CI: 0.64–4.07, *P*  = 0.31) (Fig 5). No heterogeneity among the included trials was detected (I^2^ = 0%, *P* = 0.91).

#### Retransplantation Due to HCV Recurrence

**Figure 6:**
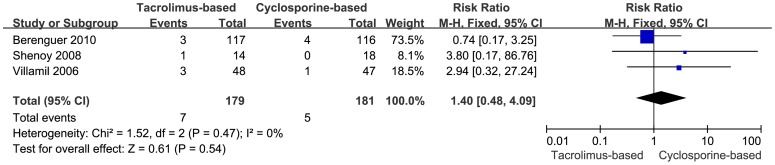
Forest plot of retransplantation due to HCV recurrence comparing tacrolimus-based to cyclosporine-based group.

Retransplantation due to HCV recurrence was reported in three trials. Seven patients in the tacrolimus-based group (n = 179) and 5 in the cyclosporine-based group (n = 181) were retransplanted due to HCV recurrence after the primary transplantation during the follow-up period. When a meta-analysis using fixed-effects model was performed concerning the incidence of retransplantation due to HCV recurrence, the outcome showed no significant statistical difference between the two groups (RR = 1.40, 95% CI: 0.48–4.09, *P* = 0.54) (Fig. 6). No heterogeneity among the included trials was detected (I^2^ = 0%, *P* = 0.47).

#### Histological HCV Recurrence

**Figure 7:**
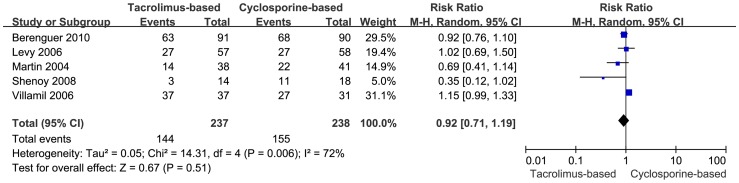
Forest plot of histological HCV recurrence included trials comparing tacrolimus-based to cyclosporine-based group.

**Figure 8:**
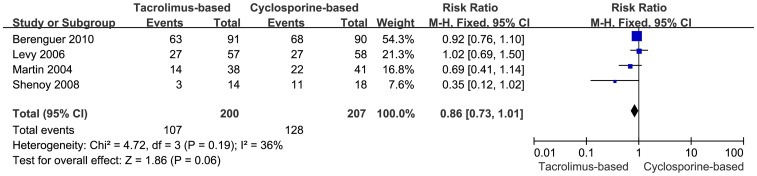
Sensitivity analysis of histological HCV recurrence.

Histological HCV recurrence was reported in five trials. A meta-analysis was performed using random-effects model, and the data referred to the number of the patients biopsied and the patients who were diagnosed as HCV recurrence by protocol biopsy. There was no significant statistical difference detected in the two groups (RR  =  0.92, 95% CI: 0.71–1.19, *P* = 0.51), while the heterogeneity was substantially significant, (I^2^ = 72%, *P* = 0.006) (Fig 7). In order to conduct a sensitivity analysis to assess the validity of outcome, the trial with highest weight was excluded. The remaining four trials, which showed 107 instances of histological HCV recurrence in the tacrolimus-based group (n = 200) and 128 instances in the cyclosporine-based group (n = 207), were included. Difference in the recurrence of HCV was not statistically significant in the two groups (RR = 0.86, 95% CI: 0.73–1.01, *P* = 0.06) (Fig 8).

### Publication bias

The funnel plots for publication bias for risk ratio in mortality show little asymmetry, but the Egger's test result was insignificant (*P* = 0.443). The result indicated no publication bias for the risk ratio pooled mortality in tacrolimus-based and cyclosporine-based groups.

## Discussion

Calcineurin inhibitors (CNIs) represent the cornerstone of immunosuppression in liver transplantation, especially tacrolimus and cyclosporine. Although in recent years many clinical trials reported the distinction between these two typical calcineurin inhibitors, there is a paucity of prospective studies comparing tacrolimus with cyclosporine in terms of their ability to reduce HCV recurrence. However, the progression of HCV-related disease is accelerated in immunosuppressed liver transplant recipients compared to immunocompetent patients, with a progressive increase in patients who have recently undergone liver transplantation [2], [14]. Hence, a meta-analysis was performed using prospective randomized studies to evaluate clinical outcomes, especially cases of HCV recurrence after liver transplantation in HCV-infected patients treated with tacrolimus-based and cyclosporine-based immunosuppression.

There was no significant statistical difference detected in terms of HCV recurrence-induced mortality, graft loss and retransplantation. In addition, the severity of histological HCV recurrence was similar in these two groups, which confirmed the outcome of a meta-analysis and some retrospective reviews [13], [15], [16]. However, two included trials [17], [18] found the mean time to histological recurrence was significantly shorter in the tacrolimus-based group. Furthermore, previous reports suggested that HCV recurrence may be more aggressive with tacrolimus therapy compared to cyclosporine microemulsion [19]. A retrospective study was established in patients who underwent liver transplantation for hepatitis C virus-induced liver disease to evaluate the impact of calcineurin inhibitors, and the cyclosporine group showed improved histological hepatitis C virus recurrence-free survival compared to the tacrolimus group (55.4% vs. 30.8% at 1 year, 18.6% vs. 10.3% at 3 years, 16.7% vs. 8.1% at 5 years, p<0.001) [20]. As for the risk factors associated with survival and histological HCV recurrence, donor age and gender combined with tacrolimus use were taken into account in previous studies [20]–[23]. Donor age was reported in only four included trials [17], [24]–[26]. The mean donor ages of these four studies ranged from 43 to 56 years, and there was no significant difference between patient populations in the two treatment groups in terms of donor age. As for the risk of the usage of tacrolimus, a randomized controlled pilot study in vivo showed that changing from tacrolimus to cyclosporine led to a modest HCV RNA drop and appeared to enhance the antiviral response of PEG/RBV [27]. Selzner et al. reported a retrospective study of 446 patients who received liver allograft for HCV-related cirrhosis; results suggested that the overall sustained virological response (SVR) was higher on CyA than on tacrolimus. Furthermore, cyclosporine improved the efficacy of the antiviral therapy in liver transplant patients compared to tacrolimus [28]. Because there is little established evidence of clinical benefits, further studies are needed to compare the therapeutic effect of CNIs in hepatitis C-infected patients after liver transplantation.

The trials included were enrolled from multiple centers, and the dosages, blood levels, durations and composition of the immunosuppressant regimens varied. In general, higher doses were used during the early post-transplantation weeks, and a gradual reduction was achieved during the first 12 months. Standard dosages of CNIs and immunosuppressant regimens have not been developed, but Barbier et al. were in favor of the minimum effective dose that would achieve reduced risk of chronic rejection in the majority of liver transplant patients [29]. In addition, conversion from a tacrolimus twice-daily formulation to a once-daily formulation was considered a safe and effective strategy for the management of stable liver transplantation patients [30]. However, minimization (reduction and withdrawal) regimens of calcineurin inhibitors were scarcely reported and remain in need of study. As for the use of steroids, the details were not reported in most of the included articles, but similar dosage of steroids was administered in both arms [17], [26], [31]. Different usage of steroids as immunosuppressant regimen might affect the clinical outcome. A meta-analysis comprising 19 RCT was conducted to evaluate the comparison of steroid-free with steroid-based immunosuppression: HCV recurrence was lower with steroid avoidance, although no individual trial reached significant statistical difference [32]. Another meta-analysis demonstrated a significant advantage of steroid-free protocols with respect to HCV recurrence [33]. However, a retrospective analysis [34] suggested that rapid tapering off of steroid dose was associated with a significantly higher rate of HCV recurrence.

More heterogeneity was detected between the trials when analyzed for histological HCV recurrence than for other clinical outcomes. The trial with highest weight was excluded so as to conduct a sensitivity analysis, and the results showed no statistically significant difference between the two groups, which was consistent with the original results that included all five trials reporting histological HCV. This may suggest that the meta-analysis outcomes were not affected by heterogeneity and their validity was acceptable.

### Limitations

Overall, several limitations of this study should be considered. The major limitation was the small number of trials available for analysis. In addition, some of the included trials were not originally designed to compare tacrolimus versus cyclosporine in hepatitis C patients after liver transplantation; rather, HCV-infected patients were considered as one subgroup in these trials. Furthermore, because of the small sample size, tests for heterogeneity were analyzed irrespective of the dosage/blood levels of different immunosuppressant agents.

The methodological quality of some included trials had medium scores, as they were not double-blind and/or the methods of randomization were not described explicitly, which might have led to exaggerated estimates of intervention benefit or contributed to discrepancies between the results [35], [36]. In other words, the heterogeneity in analyzing histological HCV recurrence might be due to the medium scores of the trials.

In addition, most authors of the included articles didn't defined graft loss in their manuscripts, and only one [26] defined graft loss as: 1) graft loss with subsequent death; 2) graft loss with retransplantation; 3) graft loss without retransplantation and loss of subsequent follow-up. In addition, the number of deaths is inferior to the number of graft loss in four manuscripts [24], [31], [37], [38]. However, the number of deaths is superior to the number of graft loss in three manuscripts [17], [25], [26]. It's a hint that the definition of graft loss in their manuscripts might be different,which may lead to different result of the meta-analysis of graft loss.

## Conclusions

In summary, our results demonstrated that HCV recurrence-induced mortality, graft loss and retransplantation, as well as the incidence of histological HCV recurrence, were not associated with the selection of different CNIs as the basic immunosuppressant in hepatitis C-infected patients.

## Supporting Information

Checklist S1 **PRISMA Checklist.**
(DOC)
